# The HALP (hemoglobin, albumin, lymphocyte, and platelet) score is associated with hemorrhagic transformation after intravenous thrombolysis in patients with acute ischemic stroke

**DOI:** 10.3389/fneur.2024.1428120

**Published:** 2024-10-25

**Authors:** Jiahao Chen, Rui Hu, Lejia Shang, Xiaoqin Li, Yisi Lin, Yu Yao, Chuanchen Hu

**Affiliations:** ^1^Department of Neurology, Affiliated Jinhua Hospital, Zhejiang University School of Medicine, Jinhua, China; ^2^Department of Neurology, Yongkang First People’s Hospital, Jinhua, China; ^3^Ruao Town Health Service Center, Shaoxing, China; ^4^Department of Neurology, The Third Affiliated Hospital of Wenzhou Medical University (Ruian People’s Hospital), Wenzhou, China

**Keywords:** acute ischemic stroke, hemorrhagic transformation, inflammation, nutrition status, predictor

## Abstract

**Background:**

Hemorrhagic transformation (HT) after intravenous thrombolysis (IVT) with rt-PA can precipitate rapid neurological deterioration, poor prognosis, and even death. The HALP score (hemoglobin, albumin, lymphocyte, and platelet) is a novel indicator developed to reflect both systemic inflammation and the nutritional status of patients. The goal of this study was to reveal the relationship between the HALP score and the risk of HT after IVT in people with acute ischemic stroke (AIS).

**Methods:**

A total of 753 patients with AIS were included in this study. Patients were divided into quartiles according to baseline HALP score. The HALP score was calculated as follows: hemoglobin (g/L) × albumin (g/L) × lymphocytes (/L)/platelets (/L). Binary logistic regression was used to reveal the connection between HALP score and HT.

**Results:**

The baseline HALP score were significantly lower in the HT than non-HT patients (*p* < 0.001). The HALP score were divided into four quartiles: Q1 (<27.4), Q2 (27.4–37.6), Q3 (37.7–49.6), Q4 (>49.6), respectively. Moreover, the severity of HT increased with decreasing HALP level (*p* < 0.001). In multivariable logistic regression, taking the Q4 as the reference, the association between Q1 and HT remained, after adjusting for confounding variables [odds ratio (OR) = 3.197, 95% confidence interval (CI) = 1.634–6.635, *p* = 0.003].

**Conclusion:**

The HALP value can predict the HT risk after IVT in patients with AIS. A lower HALP level was associated with an increased severity of HT post-IVT.

## 1 Introduction

Acute ischemic stroke (AIS) is a main cause of death and disability worldwide. Currently, in the very early stage of AIS, intravenous thrombolysis (IVT) with recombinant tissue plasminogen activator (rt-PA) is the main method of treatment, which can effectively improve functional outcomes of patients with AIS (1). However, despite improving neurological prognosis, IVT with rt-PA also increases the risk of hemorrhagic transformation (HT) (2), a common post-IVT complication with a prevalence of 27–37% (3). HT can precipitate rapid neurological deterioration, poor prognosis, and even death (4). Consequently, the accurate and convenient assessment of HT risk post-IVT in patients with AIS is paramount.

After the onset of AIS, inflammatory status plays a pivotal role in neurologic function injury and protection. Recently, neutrophil to lymphocyte ratio (NLR), an important inflammatory index, of which level has been seemed like a substantial risk factor for HT after IVT (5). In addition, platelet to lymphocyte ratio (PLR) is another inflammatory measure, is associated with HT and In-hospital mortality in AIS patients with large-artery occlusion (6). Albumin level was employed as a primary indicator for the patient’s nutritional status. In a cohort analysis, researchers discovered a link between low serum albumin levels and an increased risk of HT post-IVT (7). On this basis, we aggregated these common indications and explored the association between them and HT after IVT in this study.

A novel marker, HALP, comprising hemoglobin, albumin, lymphocyte, and platelet levels, has been developed to reflect both systemic inflammation and the nutritional status of patients (8–11). Inflammation is a known pathophysiological mechanism of HT, especially following reperfusion post-IVT (12, 13). Additionally, malnutrition is recognized as a risk factor for disability and long-term mortality in patients with AIS (14). Yet, no studies have examined the association between the HALP score and HT risk post-IVT in patients with AIS.

Therefore, this study aimed to investigate the relationship between HALP score and HT in patients with AIS following IVT treatment.

## 2 Methods

### 2.1 Patients

This retrospective study was conducted at the Affiliated Jinhua Hospital, School of Medicine of Zhejiang University. The study included all consecutive patients over 18 years who underwent IVT with rt-PA (0.9 mg/kg, maximum 90 mg) between January 2020 and January 2024. The study was approved by the Institutional Review Board and Ethics Committee of the Affiliated Jinhua Hospital, School of Medicine of Zhejiang University, with informed consent waived due to the retrospective nature of the study and the anonymity of all data.

AIS diagnosis was confirmed via CT or magnetic resonance imaging (MRI) upon admission. The IVT with rt-PA was administered within 4.5 h of symptom onset for all suitable participants, strictly adhering to the ESO guidelines (15). The exclusion criteria were as follows: (1) data unavailable; (2) absence of follow-up CT or MRI within 24 h; (3) severe diseases, including tumors and trauma; (4) suffered from infection within 2 weeks before admission.

### 2.2 Data collection

Demographic characteristics (age and gender) and medical history of atrial fibrillation (AF), diabetes mellitus (DM), hypertension, coronary heart disease (CHD), hyperlipidemia, cigarette smoking, and alcohol consumption were extracted from medical records. Based on the National Institutes of Health Stroke Scale (NIHSS), stroke severity was evaluated upon admission (16). Short-term functional outcome was assessed by modified Rankin Scale (mRS) and Barthel index (BI) score at discharge. Trial of ORG 10172 in Acute Stroke Treatment (TOAST) criteria were used to classify the AIS subtypes (17). Fasting peripheral venous blood samples were collected within 24 h after admission to obtain laboratory data, including serum albumin, hemoglobin, lymphocyte, and PLT levels. The HALP score was calculated as follows: hemoglobin (g/L) × albumin (g/L) × lymphocytes (/L)/platelets (/L).

### 2.3 Definition and classification of HT subtypes

Patients with possible AIS underwent cranial CT examination prior to IVT, and subsequent cranial CT or MRI was performed within 24 h post-IVT to screen for HT. In case of clinical symptom deterioration, an immediate imaging examination was conducted. HT was radiologically classified into four subtypes based on follow-up CT/MRIs by two experienced neuroradiologists blinded to the clinical data, following the criteria of the European Cooperative Acute Stroke Study (ECASS) (18): hemorrhagic infarction (HI)-1 (small petechiae along the periphery of the infarct), HI-2 (more confluent petechiae around the infarcted area without a space-occupying effect), parenchymal hematoma (PH)-1 (hematoma <30% of the infarcted area with a mild space-occupying effect), and PH-2 (hematoma >30% of the infarcted area with a significant space-occupying effect).

### 2.4 Statistical analyses

Data distribution normality was tested using the Kolmogorov–Smirnov test. Baseline characteristics were expressed as mean ± standard deviation, those with non-normal distributions as median with interquartile range, and categorical variables as relative frequency and percentage. Continuous variables were compared using the student’s *t*-test or the Mann–Whitney *U* test; categorical variables were compared using the chi-square test or Fisher’s exact test, as appropriate. Based on the quartiles of the HALP scores, all patients in the study were divided into four groups. One-way analysis of variance (ANOVA) or Kruskal–Wallis test was used to perform statistical comparisons of HALP score stratification for continuous variables. For significantly different variables, we employed Tukey’s honestly significant difference (HSD) test as the post-hoc test. To evaluate whether the HALP stratification was an independent predictor of HT, a multivariate-adjusted binary logistic regression was performed after adjusting for conventional confounding factors and significant variables (*p* < 0.1) identified in univariate logistic regression analysis. The predictive capacity of the HALP score in discriminating possible HT was assessed by receiver operating characteristic curves (ROC). Spearman’s rank correlation test was used to analyze the correlations between the HALP score and ECASS subtypes. Two-sided *p* < 0.05 was considered significant. All statistical analyses were performed using R for MacOS, version 4.1.2.

## 3 Results

### 3.1 Baseline characteristics

**Figure 1:**
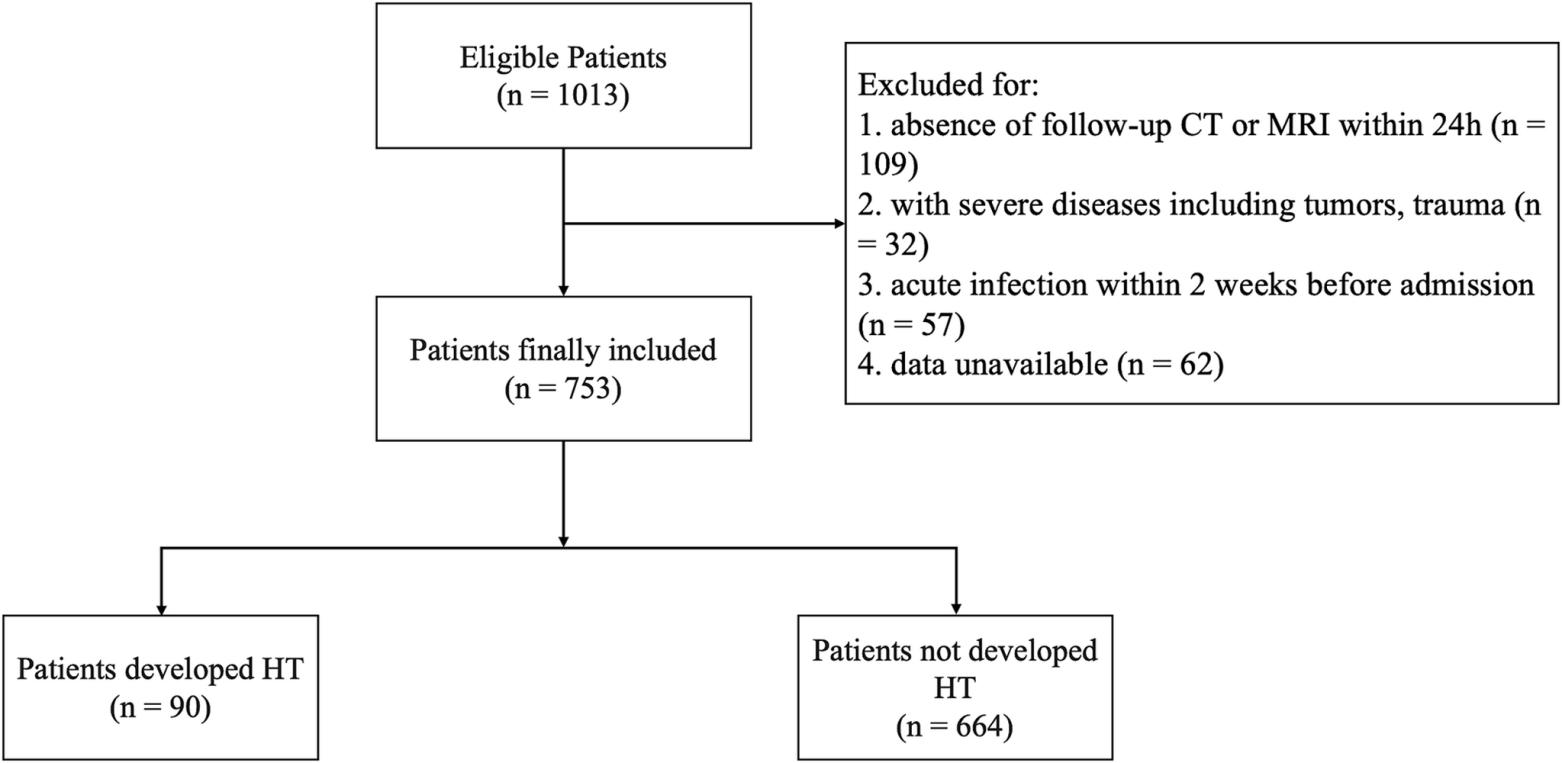
Flowchart of patient recruitment.

This study included 753 patients with AIS (Figure 1), with a median age of 70 (59–77) years; 66.7% (503) were male. Among these, 11.8% (89) developed HT, while 88.1% (664) did not.

### 3.2 Baseline characteristics of patients with and without HT

Table 1 presents the baseline characteristics and laboratory findings of the patients with AIS. The HT group had a significantly lower HALP score (29.8 versus 38.7, *p* < 0.001) compared to the non-HT group. Significant differences were also observed in age, baseline NIHSS score, mRS score at discharge, BI score at discharge, TOAST classification, AF, white blood cell (WBC) count, CRP, HDL-C, and LDL-C (*p* < 0.05). The HT group also exhibited lower hemoglobin, albumin, and lymphocyte (*p* < 0.05).

**Table 1:** Comparison of baseline characteristics between patients with or without HT.

Variables	Non-HT (*n* = 664)	HT (*n* = 89)	*p*-value
Demographics			
Age, years (IQR)	68.0 (58.0–68.0)	76.5 (69.5–83.8)	**<0.001**
Gender (male, %)	441 (66.4%)	62 (68.8%)	0.728
Vascular risk factor			
Hypertension, *n* (%)	536 (80.7%)	74 (82.2%)	0.844
DM, *n* (%)	151 (22.7%)	21 (23.3%)	1.000
AF, *n* (%)	109 (16.4%)	39 (43.3%)	**<0.001**
CHD, *n* (%)	44 (66.3%)	6 (66.7%)	1.000
Hyperlipidemia, *n* (%)	122 (18.4%)	9 (10.0%)	0.689
History of stroke, *n* (%)	99 (14.9%)	10 (11.2%)	0.445
History of smoking, *n* (%)	267 (40.2%)	28 (31.1%)	0.122
History of drinking, *n* (%)	301 (45.3%)	31 (34.4%)	0.066
Clinical information			
SBP at admission, mmHg (IQR)	152.0 (136.8–167.0)	158.0 (139.0–170.8)	0.178
DBP at admission, mmHg (IQR)	86.0 (76.0–95.0)	88.0 (73.3–97.0)	0.901
NIHSS at admission (IQR)	3.0 (1.0–5.0)	6 (3.0–11.8)	**<0.001**
mRS at discharge (IQR)	2.0 (1.0–3.0)	3.0 (2.0–5.0)	**<0.001**
BI at discharge (IOR)	80 (60–95)	50 (20–70)	**<0.001**
DNT, min (IQR)	38.0 (29.0–50.0)	39.0 (33.5–50.5)	0.257
TOAST classification			**<0.001**
Large artery atherosclerosis, *n* (%)	218 (32.8%)	27 (30.3%)	
Cardioembolism, *n* (%)	116 (17.5%)	43 (48.3%)	
Small vessel occlusion, *n* (%)	260 (39.2%)	10 (11.2%)	
Other determined, *n* (%)	6 (0.9%)	0 (0.0%)	
Undetermined, *n* (%)	64 (9.6%)	9 (10.1%)	
Laboratory signs			
Hemoglobin, g/L (IQR)	136.0 (125.0–148.0)	129.0 (117.0–141.0)	**0.002**
Albumin, g/L (IQR)	38.5 (36.5–40.5)	37.6 (34.8–40.4)	**0.032**
Lymphocyte, 10^9^/L (IQR)	1.4 (1.1–1.8)	1.1 (0.8–1.4)	**<0.001**
Platelet, 10^9^/L (IQR)	195.0 (155.0–234.0)	189.0 (141.0–233.0)	0.174
HALP (IQR)	38.7 (28.3–50.8)	29.8 (21.8–39.4)	**<0.001**
WBC, 10^9^/L (IQR)	6.7 (5.5–8.3)	7.7 (6.3–10.0)	**<0.001**
Neutrophile, 10^9^/L (IQR)	3.3 (2.3–4.7)	5.3 (3.8–7.7)	**<0.001**
CPR, mg/L (IQR)	1.2 (0.5–4.2)	6.7 (1.2–14.9)	**<0.001**
Glucose, mmol/L (IQR)	5.1 (4.6–6.1)	5.5 (4.8–6.6)	0.065
LDL, mmol/L (IQR)	2.9 (2.3–3.5)	2.6 (2.1–3.2)	**0.004**
HDL, mmol/L (IQR)	1.2 (1.0–1.4)	1.3 (1.0–1.4)	**0.027**

Data are expressed as mean ± SD, median (interquartile, range), or *n* (%) as appropriate. Comparisons among groups were performed using student’s *t*-test, or Mann–Whitney *U* test, chi-square test or Fisher’s exact test, as appropriate. Bold indicates *p* < 0.05. HT, hemorrhagic transformation; DM, diabetes mellitus; AF, atrial fibrillation; CHD, coronary heart disease; NIHSS, National Institutes of Health Stroke Scale; mRS, modified Rankin Scale; DNT, door to needle time; WBC, white blood count; CPR, C reactive protein; LDL, low density lipoprotein.

### 3.3 Baseline characteristics of patients according to HALP score quartiles

Patients were categorized into four groups based on HALP score quartiles: Q1 (<27.4), Q2 (27.4–37.6), Q3 (37.7–49.6), Q4 (>49.6). Table 2 displays demographics, vascular risk factors, clinical information, laboratory findings, TOAST classifications, and laboratory signs according to HALP quartiles. Significant differences were found in age, gender, baseline NIHSS score, mRS score at discharge, BI score at discharge, TOAST classification, history of smoking, HDL, LDL, CRP, hemoglobin, albumin, lymphocytes, and platelets (*p* < 0.05).

**Table 2:** Comparison of baseline characteristics between patients according to HALP quartiles.

Variables	HALP quartiles					
	All Patients (*n* = 753)	Quartile 1 (*n* = 188)	Quartile 2 (*n* = 189)	Quartile 3 (*n* = 188)	Quartile 4 (*n* = 188)	*p*-value
Demographics						
Age, years (IQR)	70 (59–77)	74 (65–82)	69 (61–76)	70 (57–77)	66 (54–73)	**<0.001**
Gender (male, %)	503 (66.7%)	114 (60.6%)	122 (64.6%)	125 (64.5%)	142 (75.5%)	**0.018**
Vascular risk factor						
Hypertension, n (%)	609 (80.8%)	155 (82.4%)	160 (84.7%)	145 (77.1%)	149 (79.3%)	0.254
DM, *n* (%)	171 (22.7%)	38 (20.2%)	45 (23.8%)	44 (23.4%)	44 (23.4%)	0.825
AF, *n* (%)	148 (19.6%)	43 (22.9%)	41 (21.7%)	38 (20.2%)	26 (13.8%)	0.121
CHD, *n* (%)	50 (6.6%)	11 (5.9%)	13 (6.9%)	11 (5.9%)	15 (8.0%)	0.816
Hyperlipidemia, *n* (%)	130 (17.2%)	22 (11.7%)	35 (18.5%)	33 (17.6%)	40 (21.3%)	0.093
History of stroke, *n* (%)	109 (14.4%)	34 (18.1%)	28 (14.8%)	27 (14.4%)	20 (10.6%)	0.237
History of smoking, *n* (%)	295 (39.1%)	64 (34.0%)	70 (37.0%)	82 (43.6%)	79 (42.0%)	0.200
History of drinking, *n* (%)	332 (44.0%)	73 (38.8%)	72 (38.1%)	88 (46.8%)	99 (52.7%)	**0.012**
Clinical information						
SBP at admission, mmHg (IQR)	152.0 (138.0–167.0)	156.0 (138.8–168.0)	152.0 (139.0–166.0)	151.0 (135.0–164.0)	148.0 (137.0–168.0)	0.558
DBP at admission, mmHg (IQR)	86.0 (76.0–95.0)	83.0 (72.0–94.3)	87.0 (76.0–94.0)	87.0 (76.0–95.3)	87.0 (78.8–96.0)	0.103
NIHSS at admission (IQR)	3.0 (1.0–6.0)	4.0 (1.0–8.0)	3.0 (1.0–6.0)	3.0 (1.8–6.0)	2.0 (1.0–4.5)	**0.001**
mRS at discharge (IQR)	2.0 (1.0–3.0)	2.0 (1.0–4.0)	2.0 (1.0–3.0)	2.0 (1.0–3.0)	1.0 (1.0–2.0)	**<0.001**
BI at discharge (IQR)	75.0 (50.0–95.0)	60.0 (30.0–86.3)	80.0 (50.0–95.0)	77.5 (55.0–95.0)	85.0 (60.0–95.0)	**<0.001**
DNT, min (IQR)	38.0 (29.5–50.0)	39.5 (30.0–50.3)	38.0 (29.0–51.5)	37.0 (29.0–48.5)	37.0 (29.0–50.0)	0.791
TOAST classification						**0.001**
Large artery atherosclerosis, *n* (%)	245 (32.5%)	73 (38.8%)	54 (28.6%)	69 (36.7%)	49 (26.1%)	
Cardioembolism, *n* (%)	159 (21.1%)	47 (25.0%)	45 (23.8%)	40 (21.3%)	27 (14.4%)	
Small vessel occlusion, *n* (%)	270 (35.8%)	49 (26.1%)	76 (40.2%)	61 (32.4%)	84 (44.7%)	
Other determined, *n* (%)	6 (0.7%)	1 (0.5%)	0 (0.0%)	1 (0.5%)	4 (2.1%)	
Undetermined, *n* (%)	73 (9.6%)	18 (9.6%)	14 (7.4%)	17 (9.0%)	24 (12.8%)	
Laboratory signs						
Hemoglobin, g/L (IQR)	136.0 (124.0–147.0)	123.5 (112.8–135.0)	134.0 (123.0–145.0)	138.5 (129.0–149.0)	146.0 (135.8–155.3)	**<0.001**
Albumin, g/L (IQR)	38.4 (36.4–40.5)	37.1 (35.0–39.1)	38.2 (36.0–39.9)	39.3 (37.0–40.8)	39.1 (37.3–41.5)	**<0.001**
Lymphocyte, 10^9^/L (IQR)	1.37 (1.1–1.7)	0.9 (0.7–1.1)	1.3 (1.1–1.5)	1.5 (1.3–1.8)	1.9 (1.6–2.3)	**<0.001**
Platelet, 10^9^/L (IQR)	193.0 (154.0–234.0)	204.5 (165.5–242.5)	203.0 (167.0–238.0)	193.0 (154.8–228.5)	175.5 (135.8–211.8)	**<0.001**
HALP (IQR)	37.6 (27.5–37.6)	21.1 (16.8–23.9)	32.8 (30.0–35.3)	42.5 (40.0–45.5)	60.3 (54.9–71.6)	**<0.001**
WBC, 10^9^/L (IQR)	6.9 (5.6–8.5)	6.6 (5.3–8.8)	6.5 (5.4–8.1)	6.8 (5.8–8.3)	7.4 (5.9–8.9)	**0.006**
Neutrophile, 10^9^/L (IQR)	4.8 (3.6–6.3)	5.1 (3.8–7.3)	4.6 (3.5–6.1)	4.7 (3.6–5.9)	4.8 (3.5–6.1)	0.088
CRP, mg/L (IQR)	1.4 (0.5–5.0)	3.0 (0.5–12.1)	1.7 (0.5–5.7)	1.0 (0.5–3.8)	0.9 (0.5–3.0)	**<0.001**
Glucose, mmol/L (IQR)	5.2 (4.6–6.2)	5.2 (4.6–6.1)	5.2 (4.6–6.2)	5.1 (4.6–6.6)	5.1 (4.6–6.0)	0.075
LDL, mmol/L (IQR)	1.2 (1.0–1.4)	2.8 (2.2–3.2)	2.8 (2.2–3.3)	2.8 (2.3–3.5)	3.0 (2.5–3.5)	**0.008**
HDL, mmol/L (IQR)	2.8 (2.3–3.4)	1.2 (1.0–1.4)	1.2 (1.0–1.4)	1.2 (1.0–1.3)	1.1 (1.0–1.4)	**0.034**

Data are expressed as mean ± SD, median (interquartile, range), or *n* (%) as appropriate. Comparisons among groups were performed using One-way analysis of variance (ANOVA) or Kruskal–Wallis test, chi-square test or Fisher’s exact test, as appropriate. Bold indicates *p* < 0.05. HT, hemorrhagic transformation; DM, diabetes mellitus; AF, atrial fibrillation; CHD, coronary heart disease; NIHSS, National Institutes of Health Stroke Scale; mRS, modified Rankin Scale; DNT, door to needle time.

### 3.4 The relationship between HALP score and HT after IVT

**Figure 2:**
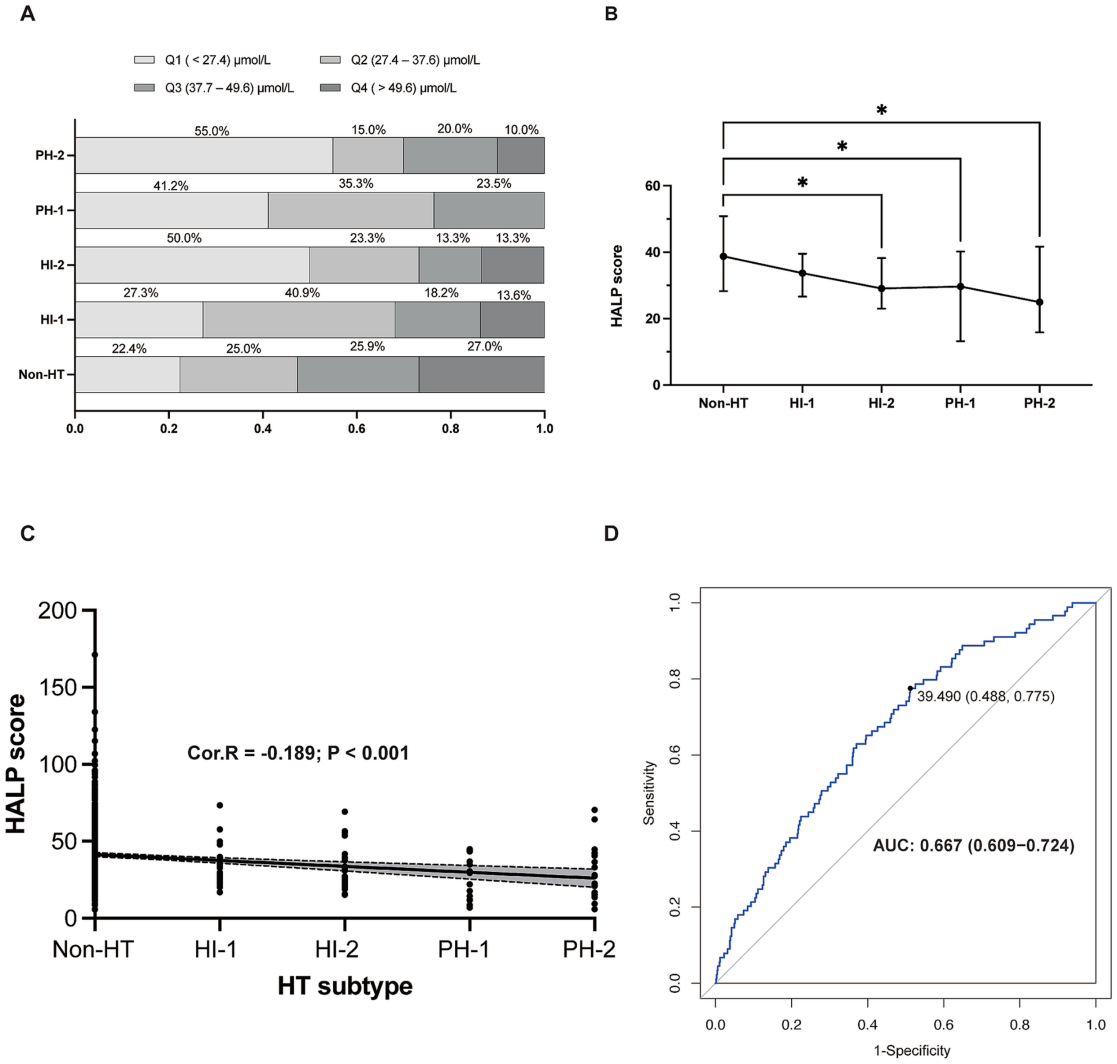
The relationship between HALP score and HT. **(A)** Proportion of patients in each HALP score with different HT subtypes. **(B)** HALP score in different subgroups of HT. **(C)** The negative relationship between HALP score and HT subtypes. **(D)** Receiver operating characteristic (ROC) curve showing the predictive ability of HALP score for HT. HALP, the hemoglobin, albumin, lymphocyte, and platelet score; HI, hemorrhagic infarct; HT, hemorrhagic transformation; PH, parenchymal hematoma; Cor. R, Spearman’s rank man’s correlation rank correlation test was test was used to analyze the correlations between HALP score and HT subtypes.

An analysis of HALP score quartiles according to HT subtype revealed that the most severe type of HT (PH-2) was significantly more prevalent in the lowest quartile (Q1) than in other types of HT or without HT (Figure 2A). The highest HALP score quartile (Q4) contained the highest proportion of patients without HT. HT severity increased with decreasing HALP score (*p* < 0.001) (Figure 2B). A negative association was found between elevated HALP score and HT severity (Spearman correlation coefficient −0.189, *p* < 0.001) (Figure 2C). The predictive capacity of the HALP score in discriminating possible HT was assessed by ROC with an area under the curve of 0.667 (0.609–0.724) (Figure 2D).

**Figure 3:**
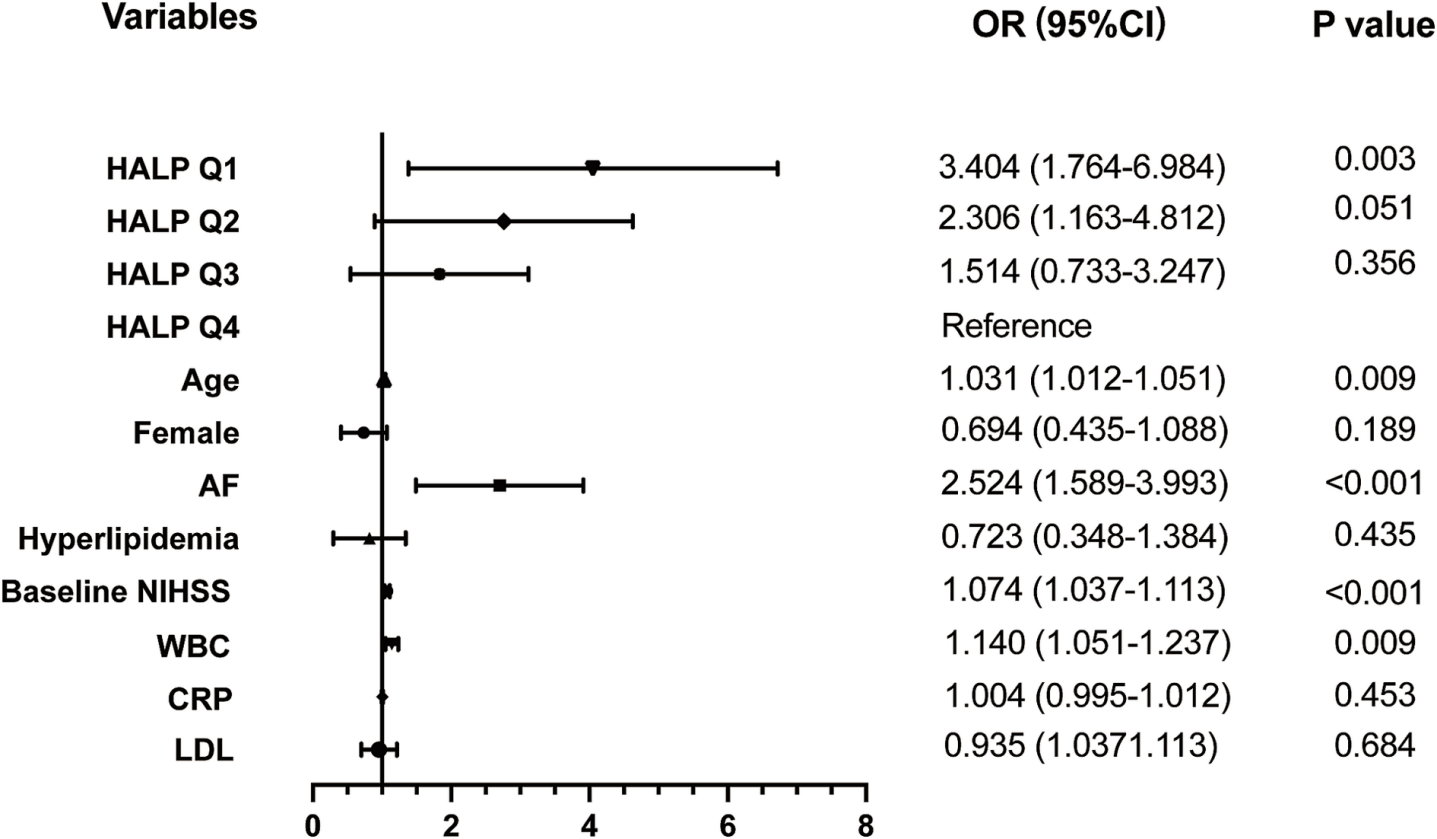
Adjusted odds ratios (OR) for the risk of HT by HALP score. HALP, the hemoglobin, albumin, lymphocyte, and platelet score; AF, atrial fibrillation; NIHSS, National Institutes of Health Stroke Scale; WBC, white blood count; CRP, C reactive protein; LDL, low density lipoprotein.

Univariate binary logistic analysis (Table 3) showed that HALP score quartiles (Q1 and Q2) were significantly associated with the risk of HT in patients with AIS (Q1: OR = 5.206, 95% CI = 2.845–10.234, *p* < 0.001; Q2: OR = 3.032, 95% CI = 1.600–6.101, *p* = 0.006). Other factors significantly associated with HT were age, AF, baseline NIHSS, WBC counts, CRP, and LDL. After adjusting for these variables, the association between Q1 and HT remained (OR = 3.197, 95% CI = 1.634–6.635, *p* = 0.003) (Figure 3).

**Table 3:** Univariate logistic regression analysis to identify relationships between variables and HT.

Variables	OR (95% CI)	*p*-value
Demographics		
Age, years	1.054 (1.037–1.073)	**<0.001**
Gender (male)	0.861 (0.570–1.279)	0.542
Vascular risk factor		
Hypertension	1.090 (0.685–1.803)	0.770
DM	0.985 (0.622–1.517)	0.955
AF	3.972 (2.680–5.970)	**<0.001**
CHD	1.019 (0.452–2.029)	0.967
Hyperlipidemia	0.439 (0.221–0.791)	**0.032**
History of ischemic stroke	0.722 (0.389–1.251)	0.357
History of smoking	0.683 (0.455–1.008)	0.114
History of drinking	0.645 (0.434–0.945)	**0.062**
Clinical information		
SBP at admission, mmHg	1.006 (0.998–1.014)	0.235
DBP at admission, mmHg	0.999 (0.987–1.012)	0.947
NIHSS at admission	1.129 (1.095–1.125)	**<0.001**
DNT, min	1.000 (0.992–1.008)	0.921
TOAST classification	0.830 (0.702–0.974)	**0.060**
Laboratory signs		
Hemoglobin, g/L	0.982 (0.972–0.993)	**0.005**
Albumin, g/L	0.947 (0.898–0.997)	0.084
Lymphocyte, 10^9^/L	0.285 (0.185–0.431)	**<0.001**
Platelet, 10^9^/L	0.997 (0.993–1.000)	0.135
WBC, 10^9^/L	1.207 (1.125–1.295)	**<0.001**
Neutrophile, 10^9^/L	1.277 (1.187–1.376)	**<0.001**
Glucose, mmol/L	0.994 (0.964–1.003)	0.551
CRP, mg/L	1.023 (1.014–1.032)	**<0.001**
LDL, mmol/L	0.640 (0.507–0.802)	**0.001**
HDL, mmol/L	1.397 (0.912–2.064)	0.168
HALP score		
HALP Q1	5.206 (2.845–10.234)	**<0.001**
HALP Q2	3.032 (1.600–6.101)	**0.006**
HALP Q3	1.850 (0.925–3.857)	0.153
HALP Q4	Ref	

HT, hemorrhagic transformation; DM, diabetes mellitus; AF, atrial fibrillation; CHD, coronary heart disease; NIHSS, National Institutes of Health Stroke Scale; mRS, modified Rankin Scale; DNT, door to needle time. The bold values were taken as statistically significant confounders as we mentioned in the “Statistical analyses” section (*p* < 0.1).

## 4 Discussion

Our study assessed the correlation between the HALP score and the risk of HT in 753 patients with AIS post-IVT. The findings indicated a significant increase in HT risk in patients with AIS after IVT with a low HALP score at admission, even after adjusting for potential and known confounders. This suggests that a low HALP score could be a potential risk factor for HT post-IVT.

HT was identified in 11.8% of all patients with AIS post-IVT, aligning with the 10–43% range reported in previous studies (19). Factors such as age, baseline NIHSS score, WBC count, and AF were independently associated with HT, corroborating previous findings (20, 21).

Initially, the HALP score was a combined scoring system predicting patient prognosis across various tumors (8–11). Recent studies have increasingly focused on the relationship between the HALP score and stroke. A Chinese study suggested that a decreased HALP score correlated with an increased mortality risk within 90 days and 1 year in a cohort of patients with AIS (22). A subsequent study in patients with cerebral venous sinus thrombosis (CVST) found that a lower HALP score was associated with a worse prognosis (23), reinforcing our findings.

Increased blood-brain barrier (BBB) permeability, particularly post-IVT, underpins post-stroke HT, accompanied by leukocyte infiltration and heightened oxidative stress (13). Brain damage can be exacerbated by a series of systemic inflammatory chemicals and cells triggered by AIS. Lymphocytes play a crucial role in resolving inflammation and tissue regeneration. Lower lymphocyte levels have consistently been associated with increased infarction volume, accelerated neurological deterioration, and unfavorable functional outcomes in patients with AIS (24). One possible mechanism is that reduced lymphocyte counts may sever as an indicator of systemic response to acute stress (25). Another suggested mechanism is that reduced lymphocyte counts means increased pre-stroke cortisol levels and sympathetic tone (26), which can secrete more pro-inflammatory cytokines that can aggravate BBB injury and finally lead to HT (27). Besides, CRP, a significant inflammation indicator (28), is typically elevated in patients with AIS. In our study, the CRP level in the HT group was higher than in the non-HT group, aligning with previous studies (29, 30). Interestingly, our study observed lower platelet levels in the HT group compared to the non-HT group, although this difference was not statistically significant (*p* = 0.174), consistent with previous studies (31, 32). This might be due to the higher prevalence of AF among patients with AIS in the HT group compared to the non-HT group. Platelets play a dual role in stroke progression, contributing to hemostasis and BBB preservation while also displaying proinflammatory effects that can exacerbate stroke outcomes (33). Recent studies have shown that inflammatory cells, including lymphocytes and platelets, play a role in cerebral ischemic injury, potentially worsening ischemic brain damage and neurological impairments (34, 35). Previous studies have established a link between decreased lymphocyte counts and increased incidence of cardiovascular disease (36). In animal models, stroke-induced immunosuppression has been shown to lead to lymphocytopenia (37). Similarly, human studies have observed a decline in lymphocyte activity within peripheral blood following a stroke (38). Regarding platelets, previous studies have shown that platelet depletion resulting from cerebral ischemia-reperfusion exacerbates brain tissue damage (39). In the acute phase of AIS, platelets guide lymphocytes to vascular injury sites, and T cells, a lymphocyte subgroup, secrete cytokines to modulate platelet activation. This process may trigger thrombo-inflammatory reactions, deteriorating tissue integrity, disrupting the BBB, and elevating oxidative stress levels (40). Hence, the acute inflammatory response is likely intricately linked with HT post-IVT.

Hemoglobin, an erythrocyte-specific protein, transports oxygen to various organs. Abnormal hemoglobin levels are associated with atherosclerosis and may pose a risk factor for AIS (41). Notably, low hemoglobin levels significantly correlate with poor outcomes and mortality post-AIS (42). Besides, a machine learning-based prediction model for HT post-IVT identified hemoglobin as a crucial indicator (43). The specific mechanism by which a low hemoglobin level is more likely to lead to HT is currently still unclear. There are several possible reasons. First, low hemoglobin level can influence energy metabolism in the infarct area, further lead to an increase in the area of infarction (42). Second, anemia could produce inflammation and oxidative stress, which could impair BBB in individuals with AIS (44). Third, coagulation disorders and extended bleeding tendencies in patients with anemia were common in patients with anemia, which were also risk factors of HT (45, 46).

Serum albumin levels serve as a useful nutritional status indicator. Studies have revealed that between 6.1 and 62% of patients with AIS are at malnutrition risk, which correlates with poor functional outcomes (47). Che et al. (7) proposed albumin as a predictor of HT post-IVT in patients with AIS and associated it with short-term good clinical outcomes, as evaluated by the BI score at 7 days. The possible mechanism may be that albumin possesses a neuroprotective effect as it can counteract oxidation, blood stasis, thrombosis, and leukocyte adhesion according to previous studies (48, 49).

HALP is a novel index reflecting the combined inflammatory and nutritional status of patients. To our knowledge, no study has reported the significance of HALP in patients with AIS undergoing IVT. The HALP score, derived from a composite calculation involving hemoglobin concentration (g/L), albumin levels (g/L), lymphocyte count (10^9^/L), and platelet count (10^9^/L), is a cost-effective and straightforward parameter for evaluating inflammation-nutrition status. This insight is crucial as immediate assessment of the inflammation-nutritional state allows neurologists to identify patients with AIS vulnerable to HT post-IVT.

This study has several limitations. First, our study was a single-centered, retrospective cohort analysis, which could not prove cause and effect. Second, the small sample size of the HT group precluded the performance of a regression analysis between the radiological HT subtypes and HALP levels. Third, HALP was measured only once upon admission, despite its potential variability pre- and post-IVT and during hospitalization. In summary, further well-designed, large-scale, prospective, multicenter cohort studies are required to validate this association.

## 5 Conclusion

In conclusion, our study revealed that the HALP value can predict the HT risk after IVT in patients with AIS. A lower HALP level was closely associated with an increased severity of HT post-IVT.

## Data Availability

The raw data supporting the conclusions of this article will be made available by the authors, without undue reservation.
